# Supplementary material for: Resolving the full spectrum of human genome variation using Linked-Reads
Source: Genome Res. 2019 Apr;29(4):635–45. doi: 10.1101/gr.234443.118 (PMC6442396; doi:10.1101/gr.234443.118)
Supplement: Supplemental Material [file supp_gr.234443.118_Supplemental_longranger-2.2.zip › longranger-2.2.tar.bz2!/lr2.2-public/lib/go/src/code.google.com/vitess/third_party/py/mock-1.0.1.tar.gz!/mock-1.0.1/html/getting-started.html]

Getting Started with Mock — Mock 1.0.1 documentation


### Navigation

- index
- next |
- previous |
- Mock 1.0.1 documentation »

# Getting Started with Mock¶

## Using Mock¶

### Mock Patching Methods¶

Common uses for Mock objects include:

- Patching methods
- Recording method calls on objects

You might want to replace a method on an object to check that
it is called with the correct arguments by another part of the system:

```
>>> real = SomeClass()
>>> real.method = MagicMock(name='method')
>>> real.method(3, 4, 5, key='value')
<MagicMock name='method()' id='...'>
```

Once our mock has been used (real.method in this example) it has methods
and attributes that allow you to make assertions about how it has been used.

Note

In most of these examples the Mock and MagicMock classes
are interchangeable. As the MagicMock is the more capable class it makes
a sensible one to use by default.

Once the mock has been called its called attribute is set to
True. More importantly we can use the assert\_called\_with() or
assert\_called\_once\_with() method to check that it was called with
the correct arguments.

This example tests that calling ProductionClass().method results in a call to
the something method:

```
>>> from mock import MagicMock
>>> class ProductionClass(object):
...     def method(self):
...         self.something(1, 2, 3)
...     def something(self, a, b, c):
...         pass
...
>>> real = ProductionClass()
>>> real.something = MagicMock()
>>> real.method()
>>> real.something.assert_called_once_with(1, 2, 3)
```

### Mock for Method Calls on an Object¶

In the last example we patched a method directly on an object to check that it
was called correctly. Another common use case is to pass an object into a
method (or some part of the system under test) and then check that it is used
in the correct way.

The simple ProductionClass below has a closer method. If it is called with
an object then it calls close on it.

```
>>> class ProductionClass(object):
...     def closer(self, something):
...         something.close()
...
```

So to test it we need to pass in an object with a close method and check
that it was called correctly.

```
>>> real = ProductionClass()
>>> mock = Mock()
>>> real.closer(mock)
>>> mock.close.assert_called_with()
```

We don’t have to do any work to provide the ‘close’ method on our mock.
Accessing close creates it. So, if ‘close’ hasn’t already been called then
accessing it in the test will create it, but assert\_called\_with()
will raise a failure exception.

### Mocking Classes¶

A common use case is to mock out classes instantiated by your code under test.
When you patch a class, then that class is replaced with a mock. Instances
are created by *calling the class*. This means you access the “mock instance”
by looking at the return value of the mocked class.

In the example below we have a function some\_function that instantiates Foo
and calls a method on it. The call to patch replaces the class Foo with a
mock. The Foo instance is the result of calling the mock, so it is configured
by modifying the mock return\_value.

```
>>> def some_function():
...     instance = module.Foo()
...     return instance.method()
...
>>> with patch('module.Foo') as mock:
...     instance = mock.return_value
...     instance.method.return_value = 'the result'
...     result = some_function()
...     assert result == 'the result'
```

### Naming your mocks¶

It can be useful to give your mocks a name. The name is shown in the repr of
the mock and can be helpful when the mock appears in test failure messages. The
name is also propagated to attributes or methods of the mock:

```
>>> mock = MagicMock(name='foo')
>>> mock
<MagicMock name='foo' id='...'>
>>> mock.method
<MagicMock name='foo.method' id='...'>
```

### Tracking all Calls¶

Often you want to track more than a single call to a method. The
mock\_calls attribute records all calls
to child attributes of the mock - and also to their children.

```
>>> mock = MagicMock()
>>> mock.method()
<MagicMock name='mock.method()' id='...'>
>>> mock.attribute.method(10, x=53)
<MagicMock name='mock.attribute.method()' id='...'>
>>> mock.mock_calls
[call.method(), call.attribute.method(10, x=53)]
```

If you make an assertion about mock\_calls and any unexpected methods
have been called, then the assertion will fail. This is useful because as well
as asserting that the calls you expected have been made, you are also checking
that they were made in the right order and with no additional calls:

You use the call object to construct lists for comparing with
mock\_calls:

```
>>> expected = [call.method(), call.attribute.method(10, x=53)]
>>> mock.mock_calls == expected
True
```

### Setting Return Values and Attributes¶

Setting the return values on a mock object is trivially easy:

```
>>> mock = Mock()
>>> mock.return_value = 3
>>> mock()
3
```

Of course you can do the same for methods on the mock:

```
>>> mock = Mock()
>>> mock.method.return_value = 3
>>> mock.method()
3
```

The return value can also be set in the constructor:

```
>>> mock = Mock(return_value=3)
>>> mock()
3
```

If you need an attribute setting on your mock, just do it:

```
>>> mock = Mock()
>>> mock.x = 3
>>> mock.x
3
```

Sometimes you want to mock up a more complex situation, like for example
mock.connection.cursor().execute(“SELECT 1”). If we wanted this call to
return a list, then we have to configure the result of the nested call.

We can use call to construct the set of calls in a “chained call” like
this for easy assertion afterwards:

```
>>> mock = Mock()
>>> cursor = mock.connection.cursor.return_value
>>> cursor.execute.return_value = ['foo']
>>> mock.connection.cursor().execute("SELECT 1")
['foo']
>>> expected = call.connection.cursor().execute("SELECT 1").call_list()
>>> mock.mock_calls
[call.connection.cursor(), call.connection.cursor().execute('SELECT 1')]
>>> mock.mock_calls == expected
True
```

It is the call to .call\_list() that turns our call object into a list of
calls representing the chained calls.

### Raising exceptions with mocks¶

A useful attribute is side\_effect. If you set this to an
exception class or instance then the exception will be raised when the mock
is called.

```
>>> mock = Mock(side_effect=Exception('Boom!'))
>>> mock()
Traceback (most recent call last):
  ...
Exception: Boom!
```

### Side effect functions and iterables¶

side\_effect can also be set to a function or an iterable. The use case for
side\_effect as an iterable is where your mock is going to be called several
times, and you want each call to return a different value. When you set
side\_effect to an iterable every call to the mock returns the next value
from the iterable:

```
>>> mock = MagicMock(side_effect=[4, 5, 6])
>>> mock()
4
>>> mock()
5
>>> mock()
6
```

For more advanced use cases, like dynamically varying the return values
depending on what the mock is called with, side\_effect can be a function.
The function will be called with the same arguments as the mock. Whatever the
function returns is what the call returns:

```
>>> vals = {(1, 2): 1, (2, 3): 2}
>>> def side_effect(*args):
...     return vals[args]
...
>>> mock = MagicMock(side_effect=side_effect)
>>> mock(1, 2)
1
>>> mock(2, 3)
2
```

### Creating a Mock from an Existing Object¶

One problem with over use of mocking is that it couples your tests to the
implementation of your mocks rather than your real code. Suppose you have a
class that implements some\_method. In a test for another class, you
provide a mock of this object that *also* provides some\_method. If later
you refactor the first class, so that it no longer has some\_method - then
your tests will continue to pass even though your code is now broken!

Mock allows you to provide an object as a specification for the mock,
using the spec keyword argument. Accessing methods / attributes on the
mock that don’t exist on your specification object will immediately raise an
attribute error. If you change the implementation of your specification, then
tests that use that class will start failing immediately without you having to
instantiate the class in those tests.

```
>>> mock = Mock(spec=SomeClass)
>>> mock.old_method()
Traceback (most recent call last):
   ...
AttributeError: object has no attribute 'old_method'
```

If you want a stronger form of specification that prevents the setting
of arbitrary attributes as well as the getting of them then you can use
spec\_set instead of spec.

## Patch Decorators¶

Note

With patch it matters that you patch objects in the namespace where they
are looked up. This is normally straightforward, but for a quick guide
read *where to patch*.

A common need in tests is to patch a class attribute or a module attribute,
for example patching a builtin or patching a class in a module to test that it
is instantiated. Modules and classes are effectively global, so patching on
them has to be undone after the test or the patch will persist into other
tests and cause hard to diagnose problems.

mock provides three convenient decorators for this: patch, patch.object and
patch.dict. patch takes a single string, of the form
package.module.Class.attribute to specify the attribute you are patching. It
also optionally takes a value that you want the attribute (or class or
whatever) to be replaced with. ‘patch.object’ takes an object and the name of
the attribute you would like patched, plus optionally the value to patch it
with.

patch.object:

```
>>> original = SomeClass.attribute
>>> @patch.object(SomeClass, 'attribute', sentinel.attribute)
... def test():
...     assert SomeClass.attribute == sentinel.attribute
...
>>> test()
>>> assert SomeClass.attribute == original

>>> @patch('package.module.attribute', sentinel.attribute)
... def test():
...     from package.module import attribute
...     assert attribute is sentinel.attribute
...
>>> test()
```

If you are patching a module (including \_\_builtin\_\_) then use patch
instead of patch.object:

```
>>> mock = MagicMock(return_value = sentinel.file_handle)
>>> with patch('__builtin__.open', mock):
...     handle = open('filename', 'r')
...
>>> mock.assert_called_with('filename', 'r')
>>> assert handle == sentinel.file_handle, "incorrect file handle returned"
```

The module name can be ‘dotted’, in the form package.module if needed:

```
>>> @patch('package.module.ClassName.attribute', sentinel.attribute)
... def test():
...     from package.module import ClassName
...     assert ClassName.attribute == sentinel.attribute
...
>>> test()
```

A nice pattern is to actually decorate test methods themselves:

```
>>> class MyTest(unittest2.TestCase):
...     @patch.object(SomeClass, 'attribute', sentinel.attribute)
...     def test_something(self):
...         self.assertEqual(SomeClass.attribute, sentinel.attribute)
...
>>> original = SomeClass.attribute
>>> MyTest('test_something').test_something()
>>> assert SomeClass.attribute == original
```

If you want to patch with a Mock, you can use patch with only one argument
(or patch.object with two arguments). The mock will be created for you and
passed into the test function / method:

```
>>> class MyTest(unittest2.TestCase):
...     @patch.object(SomeClass, 'static_method')
...     def test_something(self, mock_method):
...         SomeClass.static_method()
...         mock_method.assert_called_with()
...
>>> MyTest('test_something').test_something()
```

You can stack up multiple patch decorators using this pattern:

```
>>> class MyTest(unittest2.TestCase):
...     @patch('package.module.ClassName1')
...     @patch('package.module.ClassName2')
...     def test_something(self, MockClass2, MockClass1):
...         self.assertTrue(package.module.ClassName1 is MockClass1)
...         self.assertTrue(package.module.ClassName2 is MockClass2)
...
>>> MyTest('test_something').test_something()
```

When you nest patch decorators the mocks are passed in to the decorated
function in the same order they applied (the normal *python* order that
decorators are applied). This means from the bottom up, so in the example
above the mock for test\_module.ClassName2 is passed in first.

There is also patch.dict() for setting values in a dictionary just
during a scope and restoring the dictionary to its original state when the test
ends:

```
>>> foo = {'key': 'value'}
>>> original = foo.copy()
>>> with patch.dict(foo, {'newkey': 'newvalue'}, clear=True):
...     assert foo == {'newkey': 'newvalue'}
...
>>> assert foo == original
```

patch, patch.object and patch.dict can all be used as context managers.

Where you use patch to create a mock for you, you can get a reference to the
mock using the “as” form of the with statement:

```
>>> class ProductionClass(object):
...     def method(self):
...         pass
...
>>> with patch.object(ProductionClass, 'method') as mock_method:
...     mock_method.return_value = None
...     real = ProductionClass()
...     real.method(1, 2, 3)
...
>>> mock_method.assert_called_with(1, 2, 3)
```

As an alternative patch, patch.object and patch.dict can be used as
class decorators. When used in this way it is the same as applying the
decorator indvidually to every method whose name starts with “test”.

For some more advanced examples, see the *Further Examples* page.

### Table Of Contents

- Getting Started with Mock
  - Using Mock
    - Mock Patching Methods
    - Mock for Method Calls on an Object
    - Mocking Classes
    - Naming your mocks
    - Tracking all Calls
    - Setting Return Values and Attributes
    - Raising exceptions with mocks
    - Side effect functions and iterables
    - Creating a Mock from an Existing Object
  - Patch Decorators

#### Previous topic

Mocking Magic Methods

#### Next topic

Further Examples

### This Page

- Show Source

### Quick search


Enter search terms or a module, class or function name.

### Navigation

- index
- next |
- previous |
- Mock 1.0.1 documentation »

© Copyright 2007-2012, Michael Foord & the mock team.
Last updated on Nov 05, 2012.
Created using Sphinx 1.1.3.
